# Graphene-coated microballs for a hyper-sensitive vacuum sensor

**DOI:** 10.1038/s41598-019-41413-9

**Published:** 2019-03-20

**Authors:** Sung Il Ahn, Yong Woo Kim, Seong Eui Lee, Minjun Kim, Kyeong-Keun Choi, Jung-Chul Park

**Affiliations:** 1 Department of Chemistry Education, Pusan National University, Busan, 46241 Republic of Korea; 2 Advanced Materials Engineering, Korea Polytechnic University, Jungwang dong, Shihung, 429-793 Republic of Korea; 3 Department of Engineering in Energy and Applied Chemistry Silla University, Busan, 617-736 Republic of Korea; 4 National Institute for Nanomaterials Technology(NINT), Pohang University of Science and Technology(POSTECH), San 31, Hyoja-Dong, Nam-Gu, Pohang, 790-784 Republic of Korea

## Abstract

Reduced graphene oxide (RGO)-coated microballs of poly (methyl methacrylate) (PMMA) used for fabricating three-dimensional sensor (3D sensor), which are expected to exhibit high sensitivity compared with conventional two-dimensional (2D) sensors, were prepared using a reaction-based assembly process. The sheet resistance and transmittance of the RGO-coated balls decreased with increasing number of coatings, implying that the RGO was well adhered to the ball by the assembly method. Two types of vacuum pressure sensors using multiple balls and a single ball were fabricated using lift-off and air-blowing methods, respectively. At pressures <1 torr, the sensors showed an increased resistance value due to the bending of graphene sheets by the Van der Waals attractive force. Further, the pressure versus resistance values at the logarithmic scale showed a linear relation, with a pressure reading error <6%. Compared with the 2D sensor fabricated using RGO, the multiball sensor exhibited almost 4–5 times higher RRC value. The single-ball sensor showed reasonable reproducibility at various temperatures. Given the size and pressure reading range of the sensor, the sensitivity of the single-ball sensor at 100 °C was approximately 6,000 times greater than that of the sensor with the highest sensitivity reported in the literature. The increase in surface area and the geometric effect of the sensing part of the single-ball sensor appeared to be responsible for its abnormally high sensitivity.

## Introduction

**Figure 1:**
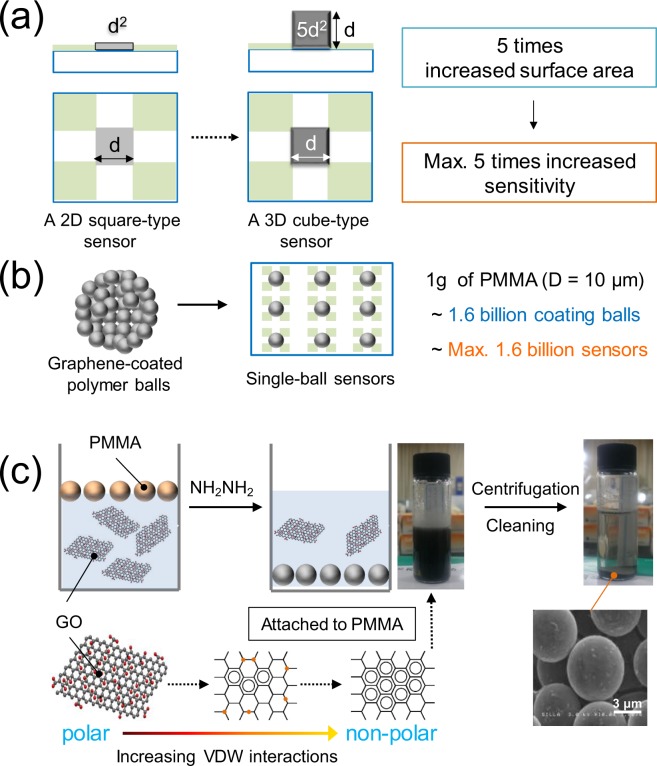
A graphic summary of the experimental concept. (**a**) A comparison of the sensing area of 2D (square) versus 3D (cube) sensors. (**b**) Theoretical number of single-ball sensors prepared from 1 g of balls with a diameter of 10 μm. (**c**) Illustration of how an RGO sheet is attached to the surface of a PMMA microball.

Graphene-based composites such as graphene-filled polymer^1^, layered graphene–polymer^2^, and polymer-functionalized graphene^3^ have been extensively researched in attempts to develop materials with enhanced properties because composite materials are commonly found to exhibit improve durability^4^, glass-transition temperature^5^, strength^2^, and thermal conductivity^6^ compared with the polymer alone. In addition, such composite materials have been widely studied as electric and electronic materials^7–11^. These studies include investigations of sensors based on interesting phenomena that have only recently been discovered. In particular, compared with conventional sensors, a sensor with a three-dimensional (3D) structure composed of a graphene–polymer composite material is expected to exhibit excellent sensing characteristics in addition to the aforementioned superior properties^10,11^. Sensors based on the steric structure of graphene–polymer composites, in particular, are expected to exhibit great advantages. However, the research on such sensors is not well established. As a simple example, when graphene-coated micropolymer balls are used as a sensor, the resultant ball sensor has a larger surface area than a two-dimensional (2D) sensor fabricated using a graphene film. Therefore, the sensor with graphene-coated balls may exhibit increased sensitivity if the sensitivity of the sensor is proportional to its surface area. As shown in Fig. 1a, assuming a sensor with a cube-shaped sensing material, the surface area of this sensor is increased by a factor of five over that of a normal 2D square sensor. Herein, the thickness of the reduced graphene oxide (RGO) film is a few tens of nanometers and can be ignored. The surface area of a single-ball sensor is increased by a maximum factor of π·(*D*/*d*)^2^ (where *D* is the diameter of the ball and *d* is the distance between electrodes in a 2D sensor). Another advantage of 3D sensors fabricated using a single-ball sensor is the number of sensors that can be manufactured from 1 g of balls (Fig. 1b). Assuming that RGO-coated poly (methyl methacrylate) (PMMA; ρ = 1.18 g/cm^3^) balls with a diameter of 10 μm are used, the number of balls and sensor devices that can be manufactured from 1 g of balls is estimated to be approximately 1.6 billion.

Although graphene polymer composites can be fabricated by simply mixing graphene and a polymer, a method of adsorbing graphene through functionalization of a polymer surface in solution is known to result in a well-defined composite material. Previous research on the reaction-based assembly process using RGO has revealed that chemical treatments are not required for attaching RGO to a polymer surface since Van der Waals (VDW) forces between RGO and the polymer surface can attach RGO sheet to the polymer, as shown in Fig. 1c ^12,13^.

In this paper, we conduct experiments from the viewpoint that an RGO sheet can be attached to the surface of PMMA balls via VDW forces and that another RGO sheet can be coated onto the RGO surface of the ball by the same method, thereby enabling control over the conductivity of the composite. We incorporated the coated balls into a pressure sensor using VDW interaction^10–12^ to investigate the influence of the coated balls on the sensitivity of the proposed 3D ball sensor.

## Results and Discussion

**Figure 2:**
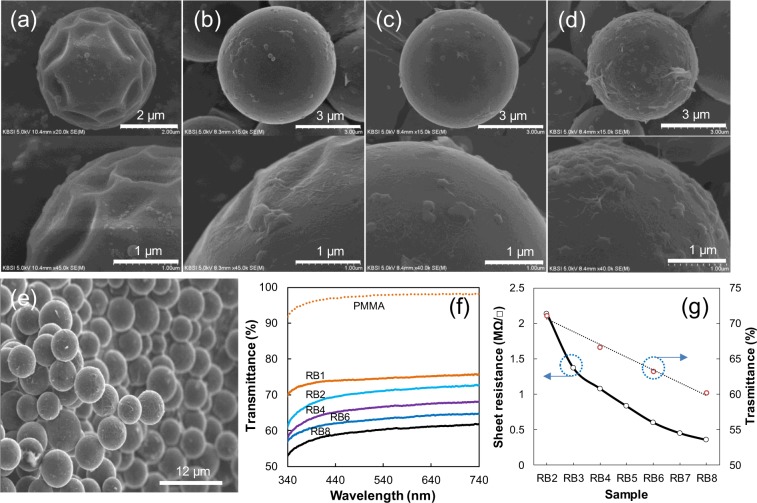
Selected FE-SEM images of RGO-coated PMMA balls: (**a**) RB2, (**b**) RB4, (**c**) RB6, (**d**) RB8, and (**e**) RB8 at different magnifications. (**f**) UV–Vis spectra (**g**) sheet resistances, and transmittance intensity at 530 nm in (**f**).

The FE-SEM images in Fig. 2a–e show the surface shape of the selected sample (sample name RB*n* implies that the composite ball is coated *n* times). The average diameter of the PMMA ball was approximately 5 μm, and some wrinkles were observed on the ball surface. The wrinkles increased slightly with increasing number of coatings. Interestingly, the FE-SEM image of the pure PMMA shows surface deformation induced by the electron beam. By contrast, with the exception of sample RB2, the surfaces of the RGO-coated PMMA samples were hardly deformed.

In the X-ray photoelectron spectroscopy (XPS) spectra, few oxygenated sites were found in heat-treated RGO samples because their proportion was reduced with increasing number of RGO coatings (see Supplementary Information Fig. S1a,b). The transmittance was measured with a UV–Vis spectrophotometer and plotted as a function of the number of coatings (Fig. 2f), revealing a continuous decrease in transmittance with increasing number of coatings, thereby indicating that the RGO layer thickness depended on the number of coatings (see also Supplementary Information Fig. S1c of Raman spectra). Figure 2g shows the sheet resistance as a function of the number of coatings, where the conductivity increases with the number of coatings. Sample RB1 exhibited an extremely high resistance, indicating that RGO was not completely coated onto its surface even when the surface area of GO added to the reaction mixture exceeded that of the PMMA (approximately 1.8 times higher). The decrease in the sheet resistance with increasing number of coatings indicates that the resistance of the balls can be adjusted for specific applications by simply controlling the number of RGO coatings.

As previously mentioned, to investigate the effect of increased surface area on the sensitivity of the sensor with a 3D sensing unit compared with that of a sensor with a 2D sensing unit, the samples were used to fabricate a vacuum pressure sensor. This vacuum pressure sensor operates on the phenomenon by which air molecules between RGO sheets escape when the vacuum is released and the distance between the sheets changes due to the VDW attraction force^13–15^. In this sensor, the change in electrical resistance is proportional to the pore volume between the RGO sheets^15^, which is directly related to the sensing surface area. Therefore, the sensitivity of the sensor improves with increasing sensing surface area. In a previous study, we reported that a decrease in pressure to approximately 1 torr decreases the overall gap between the RGO sheets, leading to a decrease in the resistance value; in addition, at pressures below 1 torr, the resistance is increased because of the bending phenomenon, where empty spaces form between RGO sheets^10^. These previous studies have shown that the reproducibility of pressure versus resistance changes is high at pressures below 1 torr. Therefore, the sensitivity change with temperature was measured in the range from 1 to 5 × 10^−3^ torr. A 2D-sensor with RGO was prepared by the same method and compared with the selected samples of RB2, RB4, RB6, and RB8. In a previous report on RGO pressure sensors, the presence of oxygenated groups in RGO was found to increase the sensitivity of the sensor because the oxygenated groups introduce voids between RGO sheets^14^. In consideration of this effect, the reference RGO was subjected to similar heat-treatment conditions (treatment at 150 °C for 12 h under air and then under vacuum) and its sensitivity was compared with that of the ball samples.

**Figure 3:**
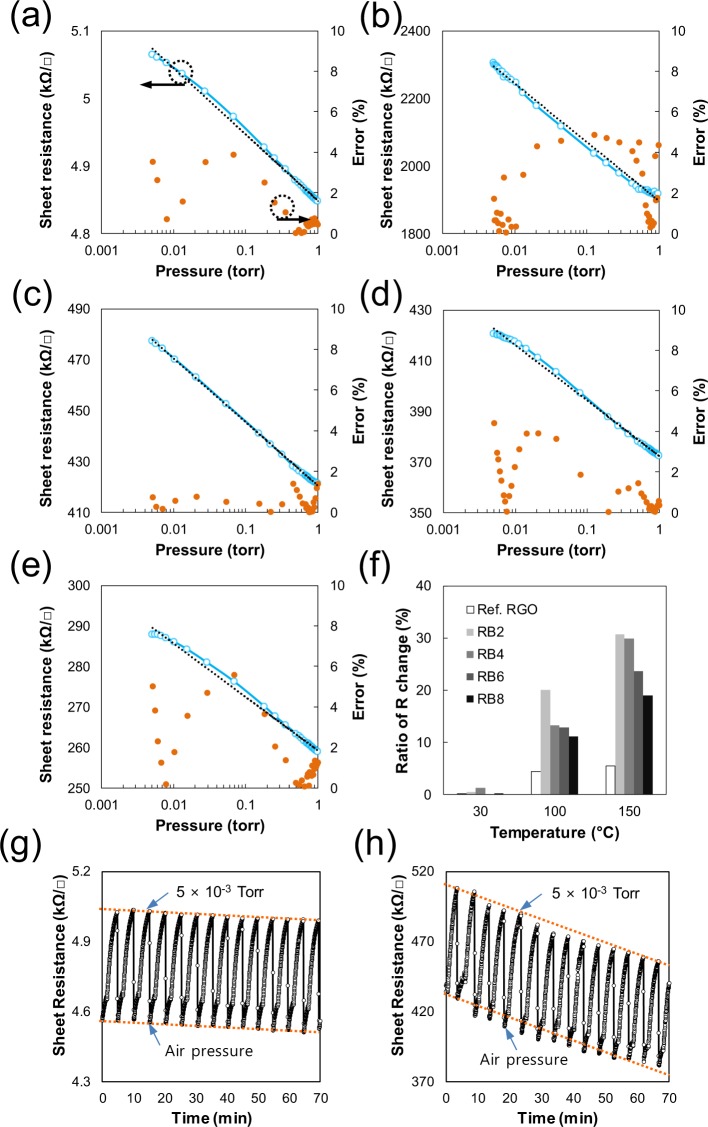
Sheet resistance of selected samples corresponding to a vacuum pressure below 1 torr measured at 100 °C (see Supplementary Information Fig. S2 for the results corresponding to 30 °C and 150 °C): (**a**) Ref. RGO, (**b**) RB2, (**c**) RB4, (**d**) RB6, and (**e**) RB8. (**f**) Sensitivities of the samples calculated using the equations in Supplementary Information Table S1. Reproducibility test results for RB6 at 100 °C compared with those for Ref. RGO: (**g**) Ref. RGO and (**h**) RB6. The percentage error was calculated as 100 × |Δ*R*_*x*_|/*R*_ideal_ (where Δ*R*_*x*_ = *R*_real_ − *R*_ideal_ at a given pressure).

As evident from the graphs in Fig. 3a–e, almost all of the samples under 1 torr exhibit an increase in resistance via the bending phenomenon of graphene sheets caused by the VDW attraction force, as previously described. In addition, the sheet resistance with respect to the logarithmic-scale pressure changes linearly. We obtained ideal lines through curve fittings of the line graphs and calculated errors using them (see Supplementary Information Table S1). As shown in Fig. 3a–e, the maximum error in the pressure readings was less than approximately 6% for all of the samples. The sensitivity of the samples was compared using the rate of resistance change (RRC) in the given pressure range:

1 $${\rm{RRC}}\,( \% )=100\times ({R}_{i}-{R}_{f})/{R}_{i}$$

where *R*_*i*_ and *R*_*f*_ are the sheet resistances at 1 and 5 × 10^−3^ torr, respectively. Figure 3f shows the RRC value for each sample, as obtained using Eq. (1). As evident in the figure, the sensitivity increases at least twofold compared with that of the 2D sensor prepared using RGO. In particular, a very large increase in sensitivity is observed with increasing measurement temperature. As mentioned in the introduction, the increase in surface area is one of the factors responsible for the high sensitivity of the sensor (other factors are noted in the discussion of the sensor fabricated with a single ball). In addition, the RRC value decreases with increasing number of coatings. This result is explained by the elastic force inside the RGO film and by the electrical conduction path of the RGO film. In a thick RGO film, the bending of the RGO sheet by the VDW attraction force occurs more at the outer surface than at the inner surface of the film where the elastic force acts largely, whereas the electrical conduction occurs mainly at a short distance near the electrode (at the inner surface). Similarly, we observe a decrease in sensitivity when measured using a thick RGO film (see Supplementary Information Fig. S3). The reproducibility of the sensor was tested by leaking and decompressing the vacuum chamber manually over a period of time. Compared with the reference RGO with a resistive signal of the same repetitive pattern, as shown in the Fig. 3g, the multiball sensor in Fig. 3h shows a continuous reduction of the resistance signal. This phenomenon appears due to the larger temperature fluctuation of RB6 sample than the RGO sensor when the measuring chamber was suddenly leaked. Because RB6 has a much larger thickness (3–5 layers of balls, each with an approximate diameter of 5 μm) than the RGO sensor (about 100 nm), sample thickness and thermal conductivity can cause the instability. The result also appears to be related to the surface area of the sample.

**Figure 4:**
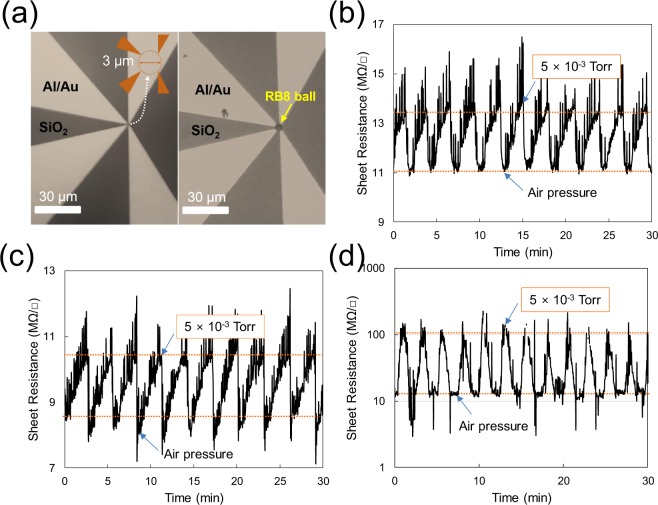
The device structure used to measure the sensor activity of a single ball and to test its reproducibility. (**a**) Microscopic images of the device used to test sensor activity (with and without a single ball). (**b**) Reproducibility test results using the device at 100 °C, (**c**) 120 °C, and (**d**) 150 °C (logarithmic scale).

Because RB8 exhibited the lowest sheet resistance, we selected this sample to fabricate a single-ball sensor. An RB8 ball was placed on a sensor device with 3 μm gaps between electrodes, as shown in Fig. 4a, using the air-blowing method (see Supplementary Information Video S1). After the thermal treatment, reproducibility tests were conducted with the multiball sensor at 100 °C, 120 °C, and 150 °C using the same measurement method. In Fig. 4b–d, all the test results show relatively clean and regular patterns of sheet resistance against a pressure change. In the case of 150 °C, the ball sensor exhibited an abnormally high RRC value of approximately 900%, likely because of electrical disconnection between the ball and the electrodes. We later found that the electrical disconnection of the ball occurred at temperatures above 130 °C due to thermal vibration of the ball. The RRC value of the single-ball sensor at 100 °C was approximately 20% and was similar to that of the multiball sensor with the highest sensitivity at the same temperature. The sensitivity of the single-ball sensor was compared with those of vacuum pressure sensors prepared using graphene or its related materials. To this end, we introduced Eq. (2) with variables of pressure range (expressed on a logarithmic scale) and device area:

2 $${\rm{Sensitivity}}=\frac{{\rm{RRC}}}{|(\mathrm{log}\,{P}_{i}-\,\mathrm{log}\,{P}_{f})|\times A}$$

where *P*_*i*_ and *P*_*f*_ are the initial and final pressures of sensor operation, respectively, and *A* is the active sensing area or the area calculated using the distance between electrodes in the sensor.

The results are summarized in Table 1^13–19^. Given the size of the sensor device and the sensing area of the sensor, the sensitivity of the single-ball sensor is approximately 1.2 × 10^−2^, which is 6000 times greater than that of the sensor reported to exhibit the highest sensitivity when operating at the resonant frequency of graphene^18^. To explain this unexpected high sensitivity, we must consider the conduction path of the charge carriers. Because a graphene-coated ball in the multiball sensor is connected to other balls, the multiball sensor has many electrical conduction paths, enabling the charge carriers to find more conductive electrical paths when the shortest path is too resistive. In the case of a single-ball sensor, few electrical conduction paths are available. Thus, the resistance of the sensor can vary greatly if a large potential barrier on the RGO sheet prevents migration of the charge carriers.

**Table 1:** Sensitivity of sensors using graphene or its related materials as an sensing materials.

Type of sensor	Dimensions (μm^2^)	Pressure range (Torr)	Sensitivity (Torr^−1^)	Sensitivity (|log P_1_/P_2_|)^−1^·μm^−2^)	Ref.
A RGO coated PMMA ball (VDW force)	**7.1**	**0.005~1**	**2.0 × 10** ^**−1**^	**~1.2 × 10** ^**−2**^	**This work (RB8 at 100 °C)**
Intercalated RGO film (VDW force)	600 × 600	0.001~1	1.1 × 10^−1^	5.3 × 10^−8^	Ahn *et al. Sci. Rep*. 2016^13^
Graphene nano-ribbon (VDW force)	600 × 600	0.001~1	5.7 × 10^−2^	1.0 × 10^−7^	Ahn *et al*., *Sci. Rep*. 2017^14^
Graphene on SiN_x_ cavity (Piezoresistivity)	490 × 490	0 (unspecified)~300	3.7 × 10^−5^	2.8 × 10^−11^	Wang *et al*., *Nanoscale*, 2016^16^
Graphene on SiO_2_/Si cavity (Piezoresistivity)	6 × 640	150~760	3.9 × 10^−6^	1.4 × 10^−9^	Smith *et al*., *Nano Lett*. 2013^17^
Graphene squeeze-film (Resonant frequency)	5 × 15	6~760	3.2 × 10^−4^	2.0 × 10^−6^	Dolleman *et al*., *Nano Lett*. 2016^18^
Graphene on SiN_x_ cavity (Piezoresistivity)	280 × 280	0 (unspecified) ~530	8.9 × 10^−6^	2.0 × 10^−11^	Zhu *et al*., *Appl. Phys*. Lett. 2013^19^

Note that the unspecified pressure was assumed to be 10^−3^ Torr.

**Figure 5:**
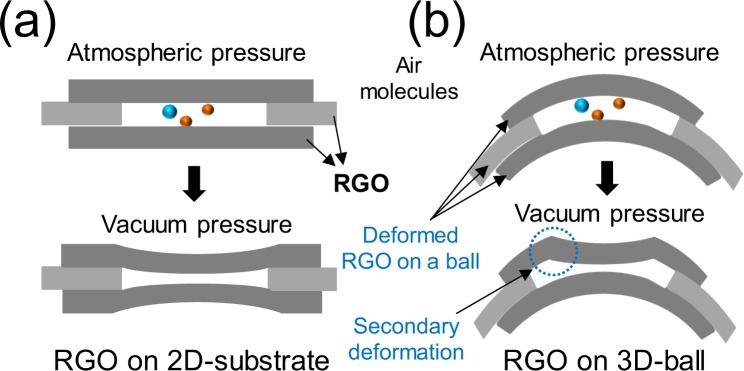
A simplified motion diagram of RGO sheets between atmospheric and vacuum pressure: (**a**) RGO sheets on a 2D substrate and (**b**) curved RGO sheets on a PMMA ball.

One of the major factors for achieving abnormally high sensitivity might be the shape of a single-ball sensor, as shown in Fig. 5. Given the size of the ball (5 μm in diameter) and the size of the RGO sheet (1 to 2 μm in this study), the RGO sheet of the ball must have a radius as large as the radius of the ball. Therefore, because of the secondary deformation of the curved RGO sheet when the vacuum is applied, the resistance change of the single-ball sensor must be larger than the resistance change of the 2D sensor.

We can also consider the vibrational effect of the PMMA ball. In Fig. 4c–d, the minimum sheet resistance of the single ball at 150 °C is higher than that at 120 °C, clearly indicating that the vibration of the PMMA ball greatly affects the resistance of the RGO-coated ball. However, a comparison of the minimum resistance at 100 °C (Fig. 4b) with that at 120 °C shows a normal resistance change corresponding to the temperature change under atmospheric conditions (see Supplementary Information Fig. S4 for resistance change against temperature change). Therefore, we assume that the vibration of the PMMA does not substantially affect the sensitivity of the single-ball sensor at temperatures below 120 °C. The number of RGO layers on the ball is likely one of the factors influencing the RRC value of the multiball and single-ball sensors. The deformed surface of the RB2 ball (coated two times) in Fig. 2a indicates that the RGO sheet cannot completely cover the PMMA surface. On the basis of this result, the number of RGO layers on RB8 balls (coated eight times) is less than eight (see Supplementary Information Fig. S7 of TEM images of RB8). With decreasing number of RGO layers or decreasing RGO film thickness, the sensitivity of the sensor increases, which explains the sensitivity of the multiball sensor (see Fig. 3f and Supplementary Information Fig. S3).

### Summary

In summary, a sensor with a three-dimensional structure of a graphene–polymer composite was expected to exhibit higher sensitivity than conventional 2D sensors. As a demonstration of this concept, RGO-coated PMMA balls were prepared by a reaction-based assembly process. Experiments showed that the desired resistance value or thickness could be controlled, as evidenced by the sheet resistance decreasing with increasing number of RGO coatings. Two types of sensors using multiple balls and a single ball were fabricated by a lift-off and air-blowing method, respectively. In the case of the multiball sensor, all of the samples showed an increase in the resistance value with the bending phenomenon of RGO sheets caused by the VDW attraction force at pressures under 1 torr. Moreover, the pressure changed linearly with the logarithm of the resistance, with an error less than 6%. The sensitivity of the multiball sensor was calculated using the RRC value in the given pressure range. Compared with the 2D-sensor using RGO, the multiball sensor had almost a 4–5 times higher RRC value at 100 °C for several reasons, including the large surface area of the sensing part in the sensor. With an increase in the number of coatings, the sensitivity of the sensor decreased, likely because the expanded conduction paths of charge carriers, and the elasticity of the RGO sheet depended on the thickness. A coated ball (RB8) was placed on a sensor device with 3 μm gaps between the electrodes, and reproducibility tests were carried out at various temperatures. Given the size and pressure reading range of the sensor, the sensitivity of the single-ball sensor at 100 °C was approximately 6,000 times greater than that of the literature-reported sensor with the highest sensitivity at the resonant frequency of graphene. Not only the increased surface area but also the geometric effect (the deformed curvature of the RGO sheet on a PMMA ball) of the single-ball sensor appeared to be responsible for its abnormally high sensitivity.

## Methods

### Preparation **o**f GO

Graphene oxide (GO) was prepared from synthetic graphite (<20 μm; Sigma-Aldrich) using the modified Hummers method^10^.

### Preparation of RGO-coated polymer balls

A 5 mL aqueous solution of 2 wt% hydrazine (80 wt%, Daejung) was dropped into 45 mL of a GO mixture comprising 0.011 wt% GO in distilled water. One gram of PMMA (~5 μm in diameter) was added to the mixture, which was then stirred for 10 h at room temperature. The resultant mixture was centrifuged, and the product was washed four times with distilled water via centrifugation. The finally obtained composite balls were dried at 60 °C for 24 h. These processes were repeated eight times to investigate whether RGO sheets could attach to the existing RGO sheet on the surface of PMMA. For each coating step, we sampled a small amount of the composite and characterized it; the samples from each step were labeled as RB1, RB2, RB3, RB4, RB5, RB6, RB7, and RB8. These samples were characterized by XPS (VG Scientific) and field-emission scanning electron microscopy (FE-SEM, S-4200, Hitachi). For UV–Vis spectrophotometry (Shimadzu, UV-2600), 0.01 g of each sample was mixed with 20 mL of distilled water containing 8% sodium dodecyl sulfate and then dispersed with bath sonicator for 10 s.

### Fabrication of a test device and measurement of pressure sensor activity

For the fabrication of a sensor using multiballs, 0.001 g of the composite sample was mixed with 1 mL of distilled water and sonicated for 10 s using a bath-type sonicator. Polyethlene terephthalate (PET) tape (50-μm thick, waterproof) with a 5-mm-diameter hole was attached to the center of the indium tin oxide electrode with a gap of 0.3 mm, and approximately 0.003 mL of the mixture was dropped into the hole. Thereafter, the sample was dried at 60 °C for 24 h, the PET tape was removed, and the film was heat-treated at 150 °C for 12 h in air and then under vacuum. In the case of a single-ball sensor, Au (0.15 μm)/Al (0.8 μm) electrodes with a 3-μm gap were fabricated on a silicon oxide (0.1 μm)/silicone wafer using a lift-off method after patterning of a photo-resistor. The detailed structures of the sensor devices are shown in Supplementary Information Fig. S5.

For investigation of the sensor activity, the sample was placed on a heating plate in a vacuum chamber with a four-point probe. The sensor properties of the selected samples (RB2, RB4, RB6, and RB8) were investigated for comparison. Before measurement, the samples were first heat-treated for 30 min at 150 °C and then the sheet resistance was measured at 30 °C, 100 °C, and 150 °C. The probe measured the sheet resistances at 1.67-s intervals against elevated pressure in vacuum under a constant pumping of approximately 0.08 torr/min until the pressure reached 5 × 10^−3^ torr. At pressures above 1 torr, we manually increased the pressure and then measured the sheet resistances of the samples.

## Supplementary information

Supporting information file
