# Supplementary material for: Graphene-coated microballs for a hyper-sensitive vacuum sensor
Source: Sci Rep. 2019 Mar 20;9:4910. doi: 10.1038/s41598-019-41413-9 (PMC6426964; doi:10.1038/s41598-019-41413-9)
Supplement: Supplementary file 1 — Supporting information file [file 41598_2019_41413_MOESM1_ESM.docx]

**Supplementary Information**

**Graphene-coated microballs for a hyper-sensitive**

**vacuum sensor**

Sung Il Ahn^1*^, Yong Woo Kim^2^, Seong Eui Lee^2*^, Minjun Kim^3^, Kyeong-Keun Choi^4^, and Jung-Chul Park^3^

^1^Department of Chemistry Education Pusan National University, Busan 46241 (Republic of Korea)

^2^Advanced Materials Engineering Korea Polytechnic University, Jungwang dong Shihung 429-793 (Republic of Korea)

^3^Department of Engineering in Energy and Applied Chemistry Silla University, Busan 617-736 (Republic of Korea)

^4^National Institute for Nanomaterials Technology(NINT), Pohang University of Science and Technology(POSTECH), San 31, Hyoja-Dong, Nam-Gu, Pohang 790-784 (Republic of Korea)

** Corresponding author*

**1. XPS and Raman spectra of RGO on PMMA balls (RB8)**

**Fig. S1.** C1s XPS spectra of RGO: a) dried at 80 °C for 24 h and b) heat-treated at 150 °C for 24 h under air and vacuum. The XPS spectra were fitted after a Shirley background correction.

c. Raman spectra of selected samples, normalized at the G band. The enlargement of the PMMA peak at 1725 cm^−1^ in the inset shows that the peak intensity decreases with increasing number of coatings. The dotted circles indicate PMMA peaks.

**2. Sheet resistance of the samples versus pressure at 30 °C and 150 °C**

**Fig. S2**. Sheet resistance of the samples versus pressure at 30 °C and 150 °C: a) Ref. RGO, b) RB2, c) RB4, d) RB6, and e) RB8. The percentage error was calculated by 100 × |Δ*R*_x_| / *R*_ideal_ (where Δ*R*_x_ = *R*_real_ – *R*_ideal_ at a given pressure).

**3. RRC value according to the thickness (resistance) of RGO**

**Fig. S3**. RRC (%) value according to the thickness (sheet resistance) of RGO: a) one-time-coated RGO (Ref.) and b) two-times-coated RGO on the sensor device.

**4. Sheet resistance against temperature**

**Fig. S4**. Sheet resistance versus temperature under atmospheric and vacuum pressure.

**5. The structure of the sensor device**

**Fig. S5.** Sensor structures: a) multiball sensor and b) single-ball sensor.

**6. Equations used in the curve fitting of Figure 2a–e and Supporting Information Figure S3**

**Table S1**. Equations for the curve fitting shown in Figure 2a–c and Supporting Information Figure S3 at the given pressure. Here, *R*_x_ is the sheet resistance at the given pressure (*P_x_*).

.

**7. TEM images of RGO on RB ball**


Figure S6. a. TEM image of sample RB8. b. Enlarged image of the red rectangle in a. c. Enlargement of the image in b (note that the yellow lines indicate the RGO layers from two balls). d. Measurement of the RGO layers using a profiler.
